# Multimodal imaging of the course of retinal changes in acute posterior multifocal placoid pigment epitheliopathy with bilateral retinal detachment: a case report

**DOI:** 10.1186/s12886-022-02624-3

**Published:** 2022-10-07

**Authors:** Gang Su, Jia Meng, Hong Li, Shan-Jun Cai

**Affiliations:** 1 Department of Ophthalmology, Affiliated Hospital of Zunyi Medical University, 149 Dalian Road, Zunyi, Guizhou 563003 China; 2 Guizhou Eye Hospital, Zunyi, China; 3 Guizhou Provincial Branch of National Eye Disease Clinical Research Center, Zunyi, China; 4 Special Key Laboratory of Ocular Diseases of Guizhou Province, Zunyi Medical University, Zunyi, China

**Keywords:** Cute posterior multifocal placoid pigment epitheliopathy, Exudative retinal detachment, Retinal pigment epithelium, Fluorescence angiography, Optical coherence tomography, Case report

## Abstract

**Background:**

To report a rare case of acute posterior multifocal placoid pigment epitheliopathy (APMPPE) with a combination of serous retinal detachment, papilledema, and retinal vasculitis.

**Case presentation:**

A 19-year-old male complained of floaters in both eyes with decreased vision for 4 days. The best corrected visual acuity of the right eye and the left eye were 1.1 and 0.9 (logMAR), respectively. In both eyes, inflammatory cells can be seen suspended within the vitreous, multiple yellow/white lesions can be seen near the macula, and retinal neuroepithelial detachment. Swelling of the optic disc with blurring of the disc margins, in the left eye. Optical coherence tomography (OCT): showed retinal detachment in both eyes. The patient received oral prednisone treatment. 1 week later, OCT showed absorption of subretinal fluid in the macula of both eyes his binocular vision improved to 0.1 (logMAR). During the subsequent 28-month follow-up, fundus fluorescein angiography and OCT revealed extensive and progressive pigment epithelial atrophy in both eyes, and abnormal retinal vascular perfusion in the right eye due to persistent retinal vasculitis. Although the patient's binocular visual acuity remained at 0.1 (logMAR).

**Conclusions:**

In the present case of APMPPE with a combination of serous retinal detachment, papilledema, and retinal vasculitis, through the multimodal imaging, further confirming that the lesions were located in the choroid, while the pigment epithelial lesions were secondary changes.

## Introduction

Acute posterior multifocal placoid pigment epitheliopathy (APMPPE) was described by Gass in 1968 [1] and is considered a self-limiting disease independent of sex, occurring in young individuals with a bilateral distribution at onset and multiple gray-white lesions in the fundus in the acute phase, which spontaneously resolve after 2–5 weeks with a clear central lesion with pigmentation and rare serous retinal detachment. APMPPE with bilateral serous retinal detachment is rare, and early reports were indistinguishable from Vogt-Koyanagi Harada disease (VKH) due to the lack of various modern imaging data [2]. Recent reports with modern imaging lack long-term follow-up and do not reflect its changing imaging presentation [3]. Here, we describe a patient diagnosed with APMPPE with a combination of bilateral serous retinal detachment, papilledema and retinal vasculitis, who was followed up for 28 months to observe the progression of retinal changes through multimodal imaging.

## Case presentation

A 19-year-old male, who complained of floaters in both eyes with decreased vision for 4 days. Ocular examination: the best corrected visual acuity (logarithm of minimal angle of resolution chart) of the right eye and the left eye were 1.1 and 0.9 (logMAR), respectively. In both eyes, inflammatory cells can be seen suspended within the vitreous, multiple yellow/white lesions can be seen near the macula, and retinal neuroepithelial detachment. The optic disc of the left eye was swollen and the edges of the optic disc were blurred. Fundus fluorescence angiography (FFA) examination (Spectralis; Heidelberg Engineering Inc., Germany): the right eye showed late venous placoid hypofluorescence in the posterior pole, and the late image showed hyperfluorescence consistent with placoid lesions and visible subretinal dye aggregation; the left eye showed posterior placoid hypofluorescence in the posterior pole corresponding to hyperfluorescence in the late image, subfoveal dye aggregation and fluorescein leakage from the optic disc. Optical coherence tomography (OCT) examination (Zeiss Model 5000, Oberkochen, Germany): showed retinal detachment in both eyes as well as intraretinal fluid accumulation located in the outer retina. Diagnosis: APMPPE in both eyes. Oral prednisone anti-inflammatory treatment was given, and visual acuity improved to 0.1 (logMAR) in both eyes after 1 week. OCT showed absorption of subretinal fluid of both eyes, and some hyperreflective signals were seen in the outer retina layer. Thereafter, the patient was followed up regularly for an additional 28 months. During the follow-up, FFA and OCT revealed extensive and progressive pigment epithelial atrophy in both eyes, and abnormal retinal vascular perfusion in the right eye due to persistent retinal vasculitis. Although the patient's binocular visual acuity remained at 0.1 (logMAR).

**Fig. 1:**
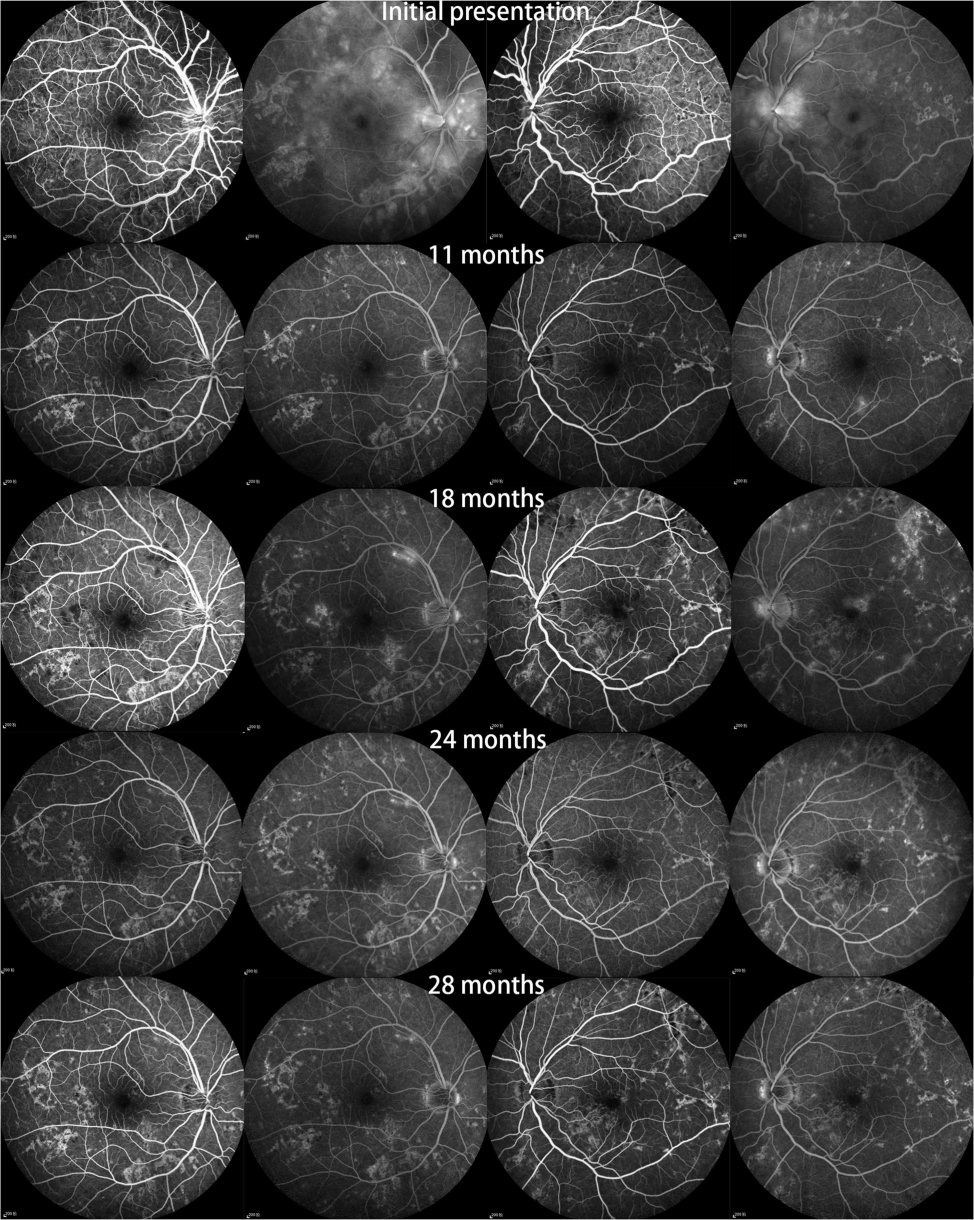
Fluorescence angiography image of the patient. The fluorescence angiogram at the early stage of the disease showed multiple placoid hypofluorescent areas in the posterior pole of the vein at the late stage, and the advanced image showed hyperfluorescence consistent with placoid lesions with subretinal dye aggregation and optic disc staining in the left eye; at the 18-month follow-up, new fluorescent changes in different locations could be seen on fluorescence angiography, and these lesions stabilized after treatment

**Fig. 2:**
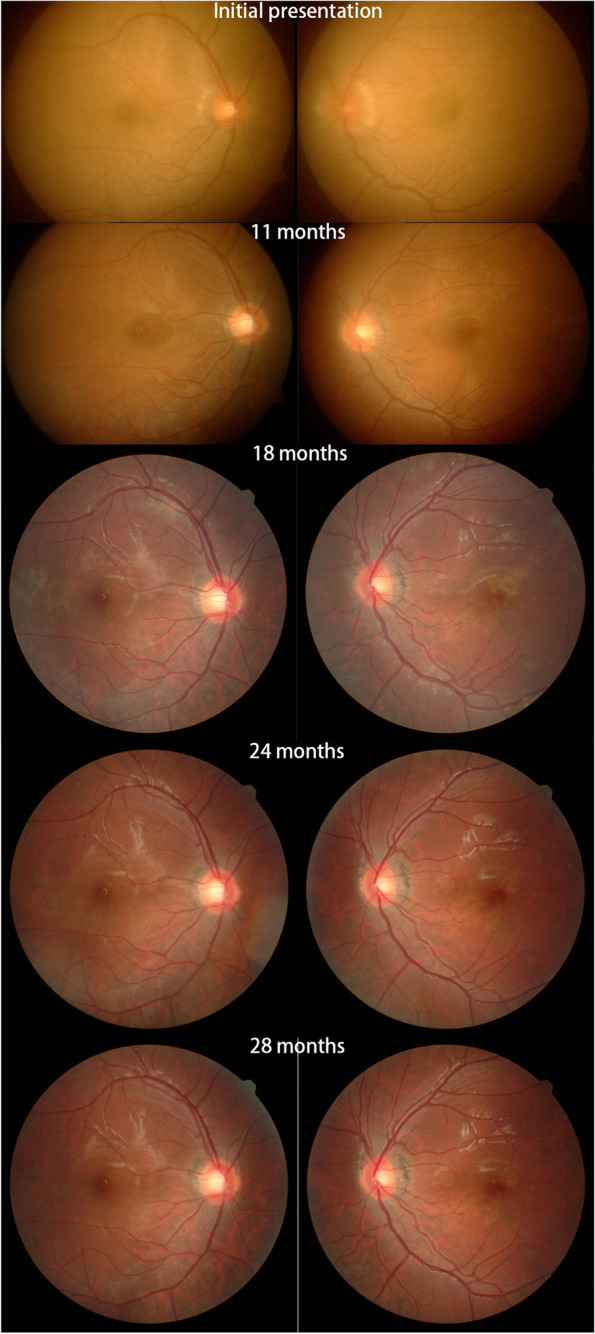
Color photograph of the patient's fundus. Retinal edema with limited neuroepithelial detachment in the early stage of the disease, with optic papilla edema in the left eye and multiple deep yellow-white lesions visible near the macula; over time, the lesions became widely distributed and pigmented

**Fig. 3:**
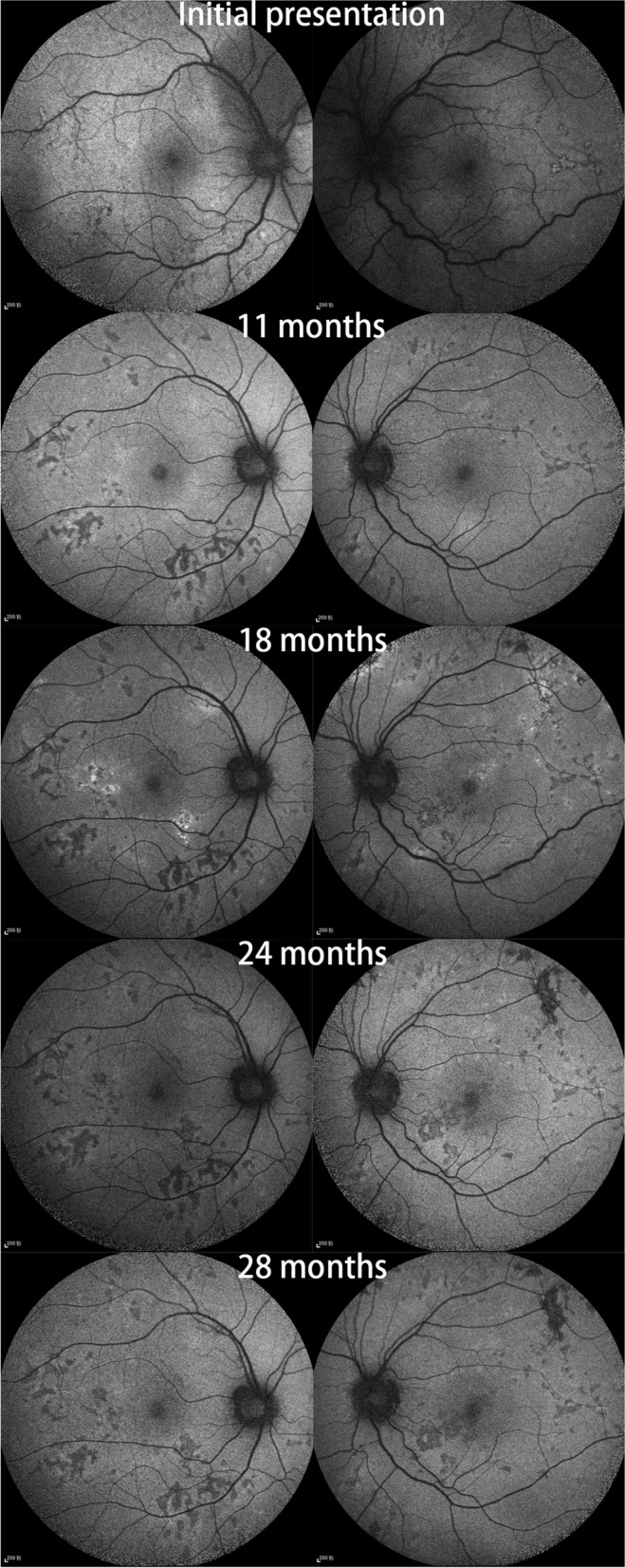
Autofluorescence images of the patient's bilateral fundus. the FAF shows a gradual increase in the extent of the lesion over the course of follow-up, with the same areas showing increased autofluorescence as the disease activity diminishes, and low autofluorescence persisting within the lesion and at the edge of the lesion. Over time, the high autofluorescence within the lesion and surrounding areas became more discrete, with a decrease in fluorescence intensity and an increase in areas of low autofluorescence

**Fig. 4:**
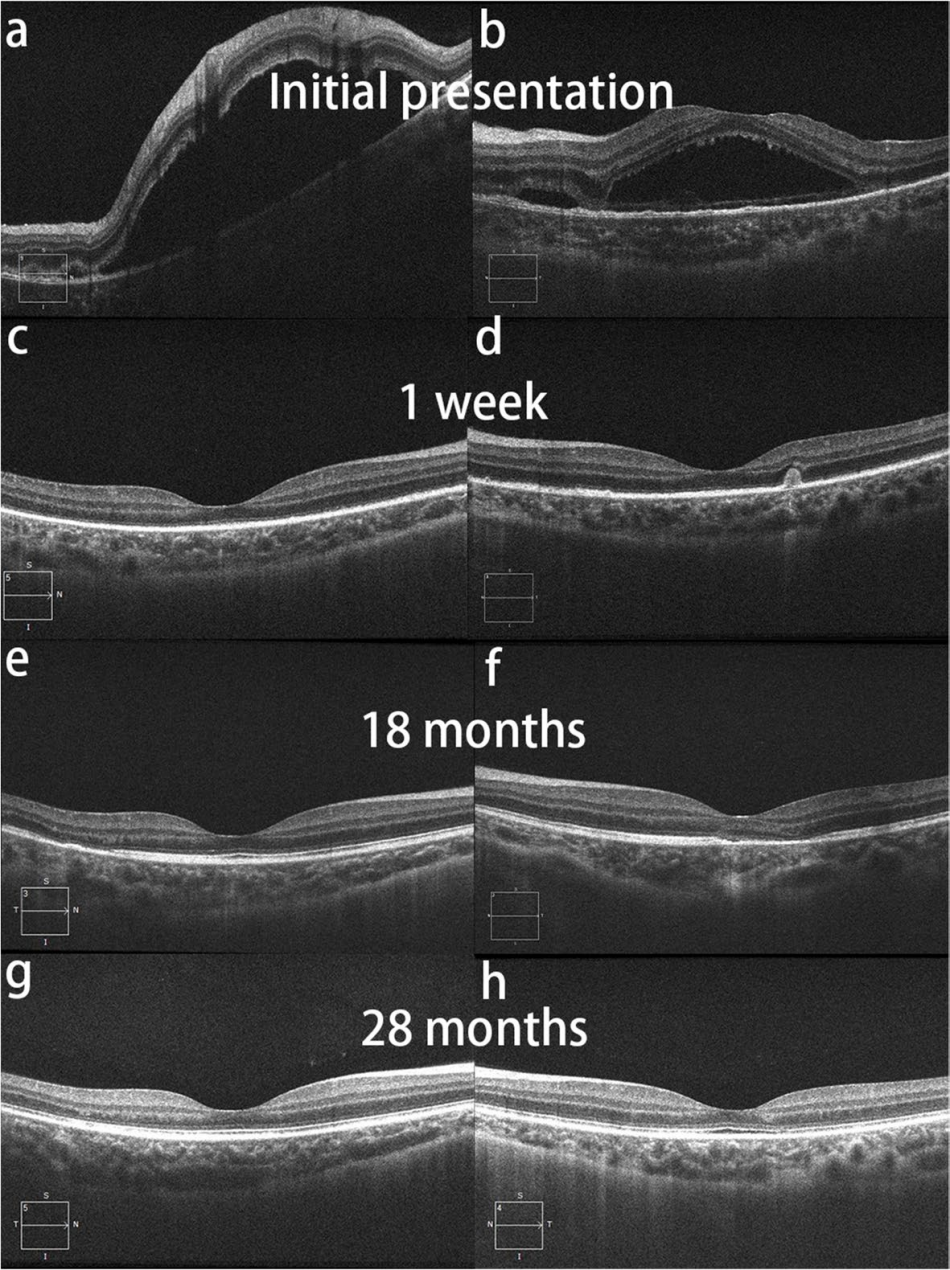
**a**, **b** OCT images of both eyes of the patient. OCT at the initial visit showed a fluid accumulating between the neurosensory retina and RPE. In addition, membranous structure and cystoid spaces were found in the left eye; **c**, **d** One week after glucocorticoid treatment, the fluid was resorbed, the inflammatory interval faded, the subretinal fluid in the macular area of the left eye was resorbed, some hyperreflective signals were visible in the outer retina, and similar retinal changes were also seen in the right eye; **e**, **f** OCT showed absorption of subretinal fluid and inflammatory deposits in the macular area of the left eye, disruption of the outer retinal strips, thinning of the outer nuclear layer, the outer plexiform layer appeared to be directly connected to the outer membrane and RPE layer, absence of the myoid band, ellipsoid band and outer photoreceptor segments, similar outer retinal lesions, also seen in the temporal side of the right eye, at 18-month follow-up visit; **g**, **h** OCT showed further reorganization and normalization of the outer retinal structures, whose outer nuclear layer had regained its normal hyporeflectivity, with partial reappearance of the ellipsoid band outer segmental membrane disc light band at the fovea, and ongoing reorganization at the connection with the RPE; the ellipsoid band was still missing in the temporal side of the right eye, but the outer retinal layer at the lesion was being reorganized, at 28-month follow-up visit

We followed this young male patient with bilateral exudative retinal detachment with APMPPE for a long time and observed the changing retinal and choroidal features by multimodal imaging. FFA of the patient at the early of onset showed late venous manifestations of multiple placoid hypofluorescent areas in the posterior pole, and late images showed hyperfluorescence consistent with placoid lesions with subretinal dye aggregation and lesions as far as the equatorial retina. Due to optic discitis in the left eye, resulting in optic disc staining (Fig. 1), localized retinal capillary dilation in the temporal periphery of the right eye, with fluorescence staining of small vessel walls; over time, the lesions continued to progress and were widely distributed, with lesions visible at different times in images of the same period (Fig. 1). The fundus photography (AFC-330; Nidek, Japan) showed that progressed from limited yellow/white lesions in the acute phase to yellow/white lesions coexistence with pigment proliferation, and widespread distribution of lesions and pigmentation over time (Fig. 2). Changes in fundus autofluorescence (FAF) were shown to lag behind the placoid APMPPE lesions seen on clinical examination during follow-up and were less numerous. As disease activity diminished, the same areas showed enhanced autofluorescence, with low autofluorescence persisting within the lesion and at the lesion margin. Over time, the high autofluorescence within the lesion and surrounding areas became more discrete, with decreased fluorescence intensity and increased areas of low autofluorescence (Fig. 3). OCT showed fluid accumulating between the neurosensory retina and RPE of this case, at the onset. After glucocorticoid treatment, the subfoveal fluid accumulation was resorbed, and some hyperreflective signals were visible in the outer retina layer, suggesting the presence of inflammatory deposits. With the absorption of inflammatory deposits, the outer retinal strips were interrupted, the outer nuclear layer was thinned, the outer plexiform layer seemed to be directly connected to the outer membrane and RPE layer, and the myoid band, ellipsoid band and photoreceptor outer segments were missing. With the prolongation of the disease, the outer retinal structures were further reorganized and normalized, the outer nuclear layer gradually regained its normal hyporeflectivity, the ellipsoidal body band at the fovea was partially reappearing with the outer segmental membrane disc light band, and the connection with the RPE was undergoing reorganization (Fig. 4).

## Discussion and conclusion

OCT features are described in the literature for both APMPPE and VKH. The typical OCT manifestations of VKH include multiple serous retinal detachment, which corresponds to late hyperfluorescence due to accumulation of fluorescein dye on FFA [4]. Serous retinal detachment caused by VKH is often accompanied by membranous structure, which divides the fluid into cystoid spaces [5]. The OCT at the beginning of this case showed retinal detachment as well as membranous structure and cystoid spaces in the left eye of the patient, and after glucocorticoid treatment, the fluid was resorbed and the inflammatory interval faded, highlighting the overlap in OCT image features between the patient with APMPPE and VKH in this case. However, the early phase FFA images of this patient showed multiple scaly hypofluorescein areas in the posterior pole of the retina, consistent with the hyperfluorescein areas in the late phase images, and no extraocular manifestations such as tinnitus and hearing impairment were seen. The patient was sensitive to glucocorticoid treatment, and the subretinal fluid was rapidly resorbed after treatment, and no sunset glow fundus changes were observed during long-term follow-up. These findings are more suggestive or in keeping with the presentation of APMPPE.

Lee et al. [3] reported a case of APMPPE with bilateral serous retinal detachment similar to our patient, but lacked long-term follow-up information. Although our case obtained good vision after treatment, fundus examination and FFA showed extensive pigmented epithelial atrophy and hyperpigmentation in the retina during the follow-up period, but new lesions appeared during the follow-up. This phenomenon has not been found in previous reports. Due to the lack of histological examination, it is unclear whether the progressive retinal pigment epithelial change of this patient is a subclinical change of the initial APMPPE as an ongoing developmental process or a result of APMPPE recurrence. At the 18-month follow-up, in addition to the retinal pigment epithelial changes, new fluorescent changes in different locations could be seen on fluorescence angiography, and these lesions stabilized after treatment. The above phenomenon is consistent with the clinical features of APMPPE. APMPPE is a monophasic disease, can recur again, but does not continue to get worse over time. This is an important distinction between VKH and APMPPE.

During the follow-up, it was found that the patient continued to develop inflammatory changes in the temporal peripheral retinal vessels of the right eye as the disease progressed, resulting in localized abnormal retinal vascular perfusion. The etiology of APMPPE is unknown and based on the patient's cold-like prodromal symptoms, it has been suggested that it is due to a viral infection and an association of adenovirus type 5 infection with APMPPE has been reported. In fact, in addition to viruses, pathogens such as tuberculosis, mycobacteria and drug allergies can be associated with the development of the disease in published case reports [6]. It has been suggested that unknown antibodies to microbial toxins may trigger an allergic reaction, which in turn leads to choroidal vasculitis, with lesions presenting as focal choroidal vasculopathy rather than primary pigment epithelial disease [7]. The recognition of persistent peripheral retinal vascular inflammatory changes based on idiopathic retinal vasculitis, more inclined to a hypersensitivity reaction triggered by antibodies to microbial toxins, which causes inflammatory changes in the choroidal and retinal vascular endothelium, which in turn leads to ischemia of the choroidal vessels and even small retinal vessels.

APMPPE is a polymorphic disease that, in addition to extensive and severe fundus involvement, exhibits a variety of associated ocular and systemic manifestations, and many associated ocular manifestations have been reported, including anterior and posterior uveitis; retinal vasculitis and papilledema; serous retinal detachment; and scleritis [8]. This polymorphism may make it difficult to differentiate between APMPPE and related clinical diseases. In the present case of APMPPE with a combination of serous retinal detachment, papilledema, and retinal vasculitis, there was considerable overlap in clinical features with VKH, and multimodality imaging played a key role in illustrating the features in both conditions. Multimodal imaging has helped to further elucidate the pathologic findings in these patients and can be used to follow clinical. During long-term follow-up, the changing pathophysiological changes were observed through multimodal imaging of the progression of their retinal changes. Vitreous opacities, serous retinal detachment, papilledema and retinal vasculitis were manifestations of choroiditis, further adding to confirm that the lesions were located in the choroid, while the pigment epithelial lesions were secondary changes.

## Data Availability

The datasets used and/or analyzed during the current study are available from the corresponding author on reasonable request.
